# Race Performance Prediction from the Physiological Profile in National Level Youth Cross-Country Cyclists

**DOI:** 10.3390/ijerph18115535

**Published:** 2021-05-21

**Authors:** Gerardo Gabriel Mirizio, Rodrigo Muñoz, Leandro Muñoz, Facundo Ahumada, Juan Del Coso

**Affiliations:** 1 Muscle Cell Physiology Laboratory, Centre of Molecular Studies of the Cell, Institute of Biomedical Sciences, Faculty of Medicine, Universidad de Chile, Santiago 8380000, Chile; gerardo.mirizio@ug.uchile.cl; 2 School of Kinesiology, Faculty of Medicine, Universidad Finis Terrae, Providencia, Santiago 7501015, Chile; roquem_08@hotmail.com; 3 Department of Investigation and Development (I + D), International Endurance Group, Córdoba 5009, Argentina; leandromartinmunoz@gmail.com (L.M.); fahumadacordoba@gmail.com (F.A.); 4 Centre for Sport Studies, Rey Juan Carlos University, Fuenlabrada, 28942 Madrid, Spain

**Keywords:** cycling, youth, power profile, anthropometry, race performance

## Abstract

Cross-country mountain biking is an Olympic sport discipline with high popularity among elite and amateur cyclists. However, there is a scarcity of data regarding the key determinants of performance, particularly in young cross-country cyclists. The aim of this study was to examine the physiological profile of youth national-level cross-country cyclists and to determine those variables that were able to best predict the performance in an official race. Ten youth cross-country cyclists of a national team underwent a complete evaluation that included anthropometric assessments, laboratory tests to evaluate the wattage at blood lactate thresholds and at maximal oxygen uptake (PO_VO2max_), and field tests to make an in-depth power profile of the athletes. The data obtained in the above-mentioned tests was analysed along with total and partial race times during a competition belonging to the *Union Cycliste Internationale* (UCI) calendar. In the present study, large and statistically significant correlations (r = −0.67 to −0.95, *p* ≤ 0.05) were found between maximal and submaximal indices of aerobic fitness and cycling performance, especially when they were normalised to body mass. A multiple regression analysis demonstrated that the wattage at 2 mmol/L, 4 mmol/L and PO_VO2max_ were able to explain 82% of the variance in total race time. In summary, the results of this study support the use of maximal and submaximal indices of aerobic power as predictors of performance in youth cross-country cyclists.

## 1. Introduction

Cross-country mountain bike competitions are one of the most challenging events at the elite level due to the high demands imposed on both the aerobic and anaerobic systems of energy [1,2,3]. Elite male cross-country cyclists have average levels of maximal oxygen uptake (VO_2max_) of around 66–78 mL/kg/min and power output values at VO_2max_ of 358–426 W (5.5–6.4 W/kg) [4,5,6,7,8], whereas females have average levels of VO_2max_ of 58–61 mL/kg/min and peak power output values of 280–320 W (4.5–5.9 W/kg) [9,10,11]. Some studies found correlations between aerobic and anaerobic fitness variables and performance in high-level cross-country cyclists, especially when such variables were normalised to body mass [7,8,12]. However, it seems that many factors are involved in mountain bike performance since, in some cases, single regression equations have not been able to explain more than 40% of the variance in race time [7]. Thus, predictive models with multiple regression analysis have been recently applied in order to predict performance from more than one prognostic variable [1,13]. This represents a better and more realistic approach in comparison with single regression models since they enable researchers to better understand the complexity of cross-country mountain biking by interconnecting the effect of multiple variables on the athlete’s physiological response.

Since the emergence of the early studies that characterised the physiological responses to cross-country races [5,10,14] and those that described cross-country cyclists’ profiles [6,7,8,11,12,15], the features of this discipline have changed dramatically. Nowadays, cross-country mountain bike races are shorter; they last about 90 min and cyclists compete in circuits of 4–6 km in length, instead of the longer circuits that were employed a few years back. Additionally, the circuits are riddled with technical sections with a variety of terrains, jumps, climbs, technical descents, rock gardens, etc., and this requires a high command of technical skills [16]. Therefore, both practitioners and researchers need more data, not only about the physiological responses to cross-country cycling events but also about the physiological and performance profiles of successful cross-country cyclists. Such information will serve to improve talent identification and enhance both training methods and the overall cross-country mountain bike performance among cyclists. Recently, some valuable data have been collected from elite mountain bikers competing in international races [3,16]. However, up to date, there is a scarcity of studies conducted on youth cross-country cyclists. The aim of this study was to examine the physiological profile of national-level youth cross-country cyclists of the Argentine National Team and to determine those variables that were able to best predict performance in an official race.

## 2. Materials and Methods

### 2.1. Participants

Ten young cross-country cyclists from the Argentinian National Team (16.3 ± 0.95 years; 67.22 ± 7.49 kg; 176.74 ± 4.23 cm) volunteered to take part in this study. At that time, the Argentinian National Junior Team was composed of ten cyclists, and therefore, this sample represents the whole group of cyclists belonging to the National Team. By the time the study was conducted, all the athletes were competing in the Junior categories of National and International championships. In the sample, there were two National champions, and two of the cyclists were runners-up in the National championship. All participants had experience in mountain bike training and competition of at least 4 years. All the individuals, as well as their parents, were informed about the benefits and risks of the research and volunteered for this study by signing an informed consent document. Protocols were approved by the local ethics committee and were in accordance with the Declaration of Helsinki for research involving human subjects.

### 2.2. Experimental Design

This is a descriptive correlational study aimed at assessing the association between laboratory- and field-based physiological testing with race performance in cross-country youth cyclists. Participants underwent a battery of measurements during three separate days, having one day in between so as to guarantee an optimal recovery between sessions. During the first session, the cyclists were subjected to a physiological assessment in the field (i.e., a power profile test) at an ambient temperature of 25 °C and relative humidity of 65%. The second and third sessions were carried out in an air-conditioned laboratory kept at 21 °C and relative humidity of 50%. During the second session, the cyclists were subjected to a graded exercise test in order to determine power at different blood lactate thresholds. After 10 min of active recovery, the cyclists performed a graded exercise in order to determine their power at VO_2max_. Finally, the athletes underwent a third session in order to conduct a complete anthropometric assessment that was then followed by evaluations of lower-limb isometric and dynamic strength performance. A week after all the physiological assessments had been conducted, the athletes competed in a *Union Cycliste Internationale* (UCI) official mountain bike competition (15.6 km, 638 m of cumulative elevation gain), and their performance was recorded for subsequent analyses and comparisons.

### 2.3. Experimental Procedures

#### 2.3.1. Pre-Experimental Standardisations

The day before the experimental sessions, participants were encouraged to avoid strenuous exercise and ingestion of stimulants (e.g., caffeine) and to sleep for at least 8 h. Fluid and diet guidelines [17,18] were given to comply with before testing to ensure carbohydrate bioavailability and euhydration. A self-selected precompetitive diet/fluid routine that fulfilled these guidelines were chosen by each cyclist before the first session, and it was replicated before the second session. On the day of each session, participants arrived at the laboratory between 10:00 and 13:00 h in a fed state (~3 h after their last meal).

#### 2.3.2. Field Physiological Assessments

In the first session, a power profile test protocol was performed in order to determine the highest power output the cyclists could maintain for a particular duration of effort in a controlled environment, in accordance with the Australian Institute of Sport [19]. For this test, participants used their own bikes, and it was performed on a flat circuit. In brief, the test started with a 5 min warm-up at 75–100 W with two sprints of 3 s at 70%–80% of peak power. Then, the cyclists performed single all-out efforts of 6, 15, 30, and 60 s with active recovery periods at 50–100 W. Finally, the test ended with two endurance efforts of 5 and 10 min, with an active recovery period at 50–100 W between them. During all these efforts, participants were encouraged to produce maximal wattage from the beginning to the end of the time established, and they were allowed to use their cycling gears to obtain an appropriate cadence. The power production and cadence (Edge 520 device plus Rally XC200 power meters, Garmin, Olathe, KS, USA) were recorded during the entire protocol with a sampling frequency of 1 s. The mean power output of the 6 s, 30 s, 60 s, 5 min, and 10 min duration exercises (P_sprint_, P_30s_, P_1min_, P_5min_, and P_10min_) was collected and analysed with specific software (version 3.5, GoldenCheetah). Critical Power (CP) was calculated based on the mean power output values of the 60 s, 5 min, and 10 min efforts. When the power output was plotted against time, the sustainable power output fell as a function of exercise duration, following an asymptotic function. This asymptote is called CP, while the curvature of the power–time relationship represents the work capacity available above the CP. The CP was calculated in accordance with the following formula [20]:

P = W’ × (1/*t*) + CP

where W’ is the fixed amount of energy in kJ above Critical Power; *t* is the time in seconds; CP is the Critical Power in watts.

#### 2.3.3. Laboratory Physiological Assessments

In the second session, participants performed two laboratory tests, carried out with their own bikes placed on an electronic indoor bike trainer (CompuTrainer, RacerMate, Inc., New York, NY, USA) and with a mobile power-measuring device (PowerTap G3, CycleOps, Madison, WI, USA). Each instrument was used in compliance with the manufacturer’s recommendations. In order to determine blood lactate thresholds, a graded exercise test was conducted first. At rest, a blood sample was obtained to assess baseline blood lactate concentration. Then, each cyclist performed a 10-min warm-up at 100 W, and then the workload was set at 150 W for 10 min. Afterwards, the load was increased by 25 W every 5 min, with 1 min of passive recovery between stages, following previous recommendations [21]. Participants were instructed to maintain a self-selected cadence during the whole test (cadence for all cross-country cyclists was between 80 and 95 rpm). At the end of each stage, blood samples were obtained from the earlobe, and the blood lactate concentration was measured using a portable lactate analyzer (Lactate Plus Meter, Nova Biomedical, Waltham, MA, USA), which had been previously calibrated. A hyperaemic lotion (Finalgon, Boehringer Ingelheim, Ingelheim am Rhein, Germany) was used to facilitate obtaining the blood samples. The heart rate was continuously monitored during the test with a chest belt and a heart rate monitor (EDGE 520, Garmin, Olathe, KS, USA). To assess the athlete’s perception of fatigue, a Rating of Perceived Exertion scale (Borg Scale 1–10 point) was used at the end of each stage. The test ended once an exponential increase in blood lactate concentration was detected or when lactate values exceeded 4 mmol/L. The blood lactate values were analysed with Lactate-E software in order to determine the power at: Lactate Threshold 1 (PO_LT1_), Lactate Threshold 2 (PO_LT2_), fixed blood lactate concentration of 2 mmol/L (PO_X2mmol/L_), fixed blood lactate concentration of 4 mmol/L (PO_X4mmol/L_), and the lactate threshold calculated by the D_max_ method (PO_Dmax_) [22]. Briefly, the PO_LT1_ corresponds to the work rate preceding an increase in blood lactate concentration of 1 mmol/L above the concentration at baseline. PO_LT2_ represents the work rate corresponding to the point of maximum acceleration of the estimated underlying lactate curve (i.e., the maximum of the second derivative of the lactate curve). PO_Dmax_ corresponds to the work rate corresponding to the point that yields the maximum perpendicular from a line L, joining the first and last lactate measurements to the estimated lactate curve [23].

After 10 min of active recovery at a power of 100 W, the athletes performed a graded exercise in order to determine their power output at VO_2max_ (PO_VO2max_). The graded exercise started at two steps (50 W) below the power that produces a blood lactate concentration of ~4 mmol/L, and then the load was increased by 25 W every 1 min, without recovery between stages. During this test, participants chose a self-selected cadence during the whole test. The test ended when volitional exhaustion was achieved or when power could not be maintained by the athlete any longer (i.e., cadence < 50 rpm). Both the total workload and the last completed stage were recorded to calculate maximal aerobic power in accordance with the equation by Kuipers et al. [24], as follows:

PO_VO2max_ = W_f_ + (*t*/60 × 25), (1)

where W_f_ is the last completed workload in watts, and *t* is the time of the uncompleted stage in seconds.

All the athletes were verbally encouraged to reach their maximal performance during the test. The test was considered valid when participants rated their perceived exertion to be higher than 9 on the 1-to-10 point Borg scale, and heart rate was higher than 80% of the age-adjusted estimate of maximal heart rate [25].

#### 2.3.4. Anthropometric Profile and Maximal Isometric Strength

The anthropometric assessments were conducted in the third session by two Level 2 anthropometrists trained by the International Society for the Advancement of Kinanthropometry (ISAK), in accordance with the ISAK protocol [26]. Body composition was calculated by applying the five-way fractionation method, which partitions the body into five anatomically defined tissue masses: adipose, muscle, residual, skeletal and skin [27]. The fractional mass obtained from the five-way fractionation method was estimated from direct anthropometric measures and expressed in absolute (kg) and relative (% of total body mass) terms. The sum of six skinfolds index (Σ6 Skf = triceps + subscapular + suprailliac + thigh calf + abdominal + medial calf) was also calculated. After the anthropometric measurements, the maximal isometric strength of the lower limbs was assessed by means of a unilateral knee extension exercise protocol, in accordance with Requena et al. [28]. The unilateral knee extension isometric force of the right and left legs was recorded with a standard calibrated strain-gauge transducer (WinLaborat, Buenos Aires, Argentina) mounted inside a metal frame, which was placed using a Velcro belt around the distal part of the ankle, above the malleoli. During the testing, the cyclists were asked to exert an isometric knee extension against the belt of the strain-gauge transducer as forcefully as possible for 2–3 s. The force-time curve was analysed on a personal computer, and the highest force value of the three maximal attempts was considered as the maximal isometric force. The dynamic strength performance of the lower limbs was assessed by means of a squat jump, in accordance with Jimenez-Reyes et al. [29]. In brief, the cyclists were asked to stand in a semi-squatting position on a contact mat (WinLaborat, Buenos Aires, Argentina) placed at ground level for each repetition. The knee angle of 90° was previously determined with a handheld goniometer. On command, participants jumped vertically, extending their hips as fast and forcefully as possible to reach a full extension of 180°, while the trunk was kept as straight as possible. Their hands remained on their hips for the entire duration of the movement. Three attempts were performed with two minutes of recovery between them. The data were recorded for the analysis of vertical jump height based on flight time.

#### 2.3.5. Cross-Country Mountain Bike Competition

The mountain bike competition consisted of four laps of 3.9 km length for a total of 15.6 km and 638 m of cumulative elevation gain. On the week before the race, all participants underwent a controlled tapering microcycle to enable a full recovery. Before the race, all the athletes carried out a 30-min warm-up in a cyclo-stimulator. All the athletes’ bikes were of similar characteristics, with carbon frames and double suspension. During the race, the athletes were allowed to drink water and sports drink ad libitum. All the athletes consumed a sports gel (25 g carbohydrate + 50 mg sodium) in each lap to standardize the intake of exogenous carbohydrates. The time to complete the race as well the time for each lap were recorded and used as performance variables.

### 2.4. Statistical Analysis

The values are expressed as means ± standard deviation (SD) in absolute values and relative to body mass. For the correlation coefficient calculation, a Spearman’s rho (ρ) statistic and its 95% confidence intervals (95% CI) were used since it is more robust and has been recommended when the data do not come from a bivariate normal distribution. The assumption of normality was verified using the Shapiro–Wilk test. For the multiple regression analysis, a typical model given by y = a + b_1*×*1_ + b_2×2_ + …b_n×n_ was used. All physiological variables with *p* < 0.05 were included, and a regression model with backward elimination was used to filter out redundant variables. By using a threshold of 5 points in the variance inflation factor, we avoided multicollinearity. The variables that were retained in the final backward elimination model were then analysed with a multivariate regression model using forward selection. Collected data from all the sources and variables were exported to an R script and analysed with RStudio statistical software [30]. Statistical significance for all analyses was set at *p* < 0.05.

## 3. Results

The cyclists’ anthropometric, physiological, and strength profiles are shown in Table 1 and Table 2.

**Figure 1:**
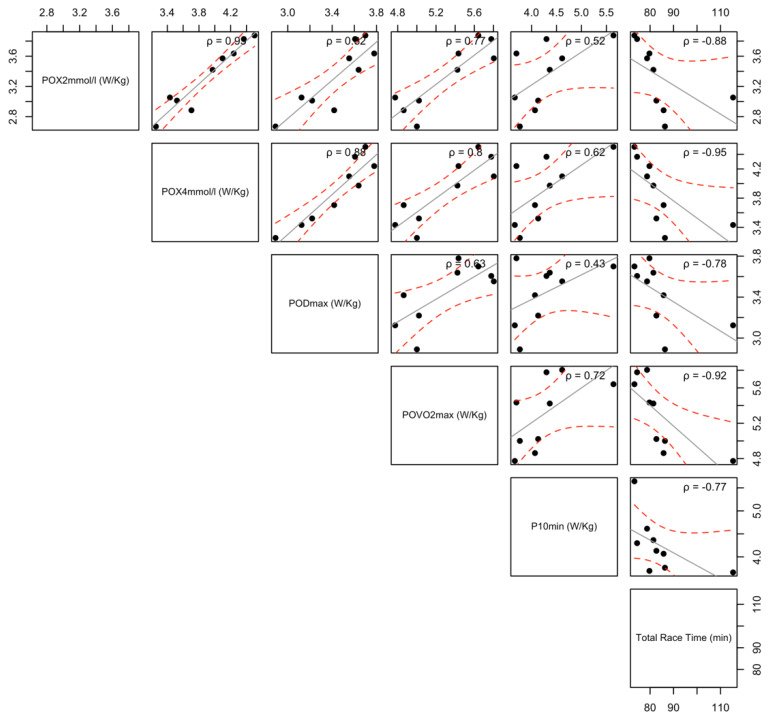
The correlation of the main physiological parameters and the total race time in elite youth cross-country cyclists.

None of the anthropometric variables or squat jump height or knee maximal isometric force correlated with race performance. However, a significant correlation between squat jump height and P_sprint_ (ρ = 0.74, *p* < 0.05, 95% CI [−0.14, 1.00]) was found. Aiming to predict race performance with a multiple regression model, we used all the physiological variables assessed in the laboratory and/or in the field and chose the variables that correlated with the total race time (all variables presented in W/kg): PO_X2mmol/L_ (ρ = −0.88, *p* < 0.01, 95% CI [−1.00, −0.39]), PO_X4mmol/L_ (ρ = −0.95, *p* < 0.001, 95% CI [−1.00, −0.72]), PO_VO2max_ (ρ = −0.92, *p* < 0.001, 95% CI [−1.00, −0.52]), P_5min_ (ρ = −0.67, *p* < 0.05, 95% CI [−0.98, −0.10]), and P_10min_ (ρ = −0.77, *p* < 0.05, 95% CI [−1.00, −0.16]), PO_Dmax_ (ρ = −0.78, *p* < 0.01, 95% CI [−0.98, −0.26]), and CP (ρ = −0.68, *p* < 0.05, 95% CI [−1.00, −0.04]). Histograms of distribution frequency, linear regression curves, and rho correlation coefficients of the top-five variables that best correlated with race time are shown in Figure 1.

For the prediction of race performance, a multiple regression analysis was conducted to assess the overall contribution of physiological variables to the interindividual variability of the total race time. As a result, a significant regression equation was found (F _(3,5)_ = 7.52, *p* = 0.03), with an R^2^ = 0.82. A participant’s predicted total race time was equal to:

Race time (min) = 255.86 − 32.87 × PO_VO2max_ − 51.18 × PO _X4mmol/L_ + 60.77 × PO _X2mmol/L_.

## 4. Discussion

The main aim of this study was to examine the physiological profile of elite youth cross-country cyclists from a National Team and to determine those variables that were best able to predict performance in an official mountain bike race. In addition to this aim, the current study provides reference values on the physiological and anthropometric profiles of elite youth cross-country cyclists who compete at national and international levels. The most important finding of this study was that 82% of the variance in race time during an official competition could be predicted by using cycling power outputs at the lactate thresholds of 2 and 4 mmol/L and the power at VO_2max_. Interestingly, anthropometric variables and maximal lower limb performance measured by a squat jump and an isometric test did not correlate with race time. Collectively, all this information suggests that the use of maximal (power output at VO_2max_) and submaximal indices (power output at lactate thresholds) of aerobic performance may be used as fair predictors of performance in youth cross-country cyclists.

### 4.1. Anthropometric Profile

In order to contrast our results with those of other studies, we summarised the anthropometric profile of elite and young cross-country cyclists reported in previous studies (Table 3). The current study revealed that the anthropometric profile of our youth cyclists was similar to that of elite cross-country cyclists. In brief, the mean height of male elite mountain bikers is between 175–180 cm [2,4,5,6,7,8,10,11,31], and it was similar to our youth male cyclists’ mean height, which was 177 ± 4 cm. The reported average body mass of elite and high-level cross-country male cyclists is between 65–72 kg [2,4,5,6,7,8,10,11,31]. Our youth male athletes weighed 67 ± 7 kg, a value within the body mass range of high-level cross-country male cyclists. These data not only show the existence of a specific anthropometric profile of cross-country cyclists but also suggest that, in regard to elite cyclists, the anthropometric profile is already achieved at an early age. It is widely assumed that body composition is a very relevant factor in elite mountain bikers. In fact, an association has been suggested between body composition and competitive levels [2]. The average percent of body fat in elite mountain bikers is between 5.3% and 14.3% [6,8,31,32]. However, our data show that elite youth cross-country cyclists have mean values of the adipose mass of 23 ± 3%, which is considerably higher. This is not surprising since adiposity values estimated through the five-way fractionation method derive from anatomically defined adipose tissue, as opposed to chemically defined fat mass estimated by traditional two-way fractionation methods [33]. Thus, whereas the five-way fractionation model evaluates adipose tissue (which comprises a lipid fraction, water, proteins, and electrolytes), the two-way fractionation models assess fat mass (which only includes the lipid fraction of adipose tissue) [34]. Indeed, when the five-way fractionation method was used to assess body composition in elite male and female youth triathletes, very similar values of adiposity to the ones reported here were observed [35]. Finally, to our knowledge, there are no studies that have conducted an in-depth anthropometric analysis of youth cross-country cyclists. In this regard, the present work aims to provide reference values of muscle, bone, and adipose tissue masses as well as of the sum of six skinfolds to be used for training purposes. Last, it is relevant to note that, despite the assumed importance of body composition for mountain bike performance [2], none of the anthropometric variables correlated with race time. This suggests that, once the appropriate body composition has been achieved in young cross-country cyclists, small interindividual variations do not explain mountain bike performance.

### 4.2. Physiological Profile

Junior mountain bike competitions have a lap length of 4–6 km and an average climbing altitude of about 500–1000 m [3], which demands a significant contribution of aerobic metabolism to fulfil the energy requirements [2]. In fact, maximal and submaximal indices of aerobic capacity, such as VO_2max_ and peak power output at the lactate threshold, have been able to explain up to 80% of the variance in off-road cycling performance [7,8]. In the present study, large correlations (ρ = −0.67 to −0.95) were found between physiological markers of aerobic fitness and cross-country cycling performance (Figure 1), as was observed in other studies involving competitive and top-level cross-country cyclists [1,8,12,13]. In accordance with previous reports, our results suggest that the relationship between physiological parameters derived from power profile tests and cross-country cycling performance is higher when the cycling power output is normalised to body mass. This highlights the importance of the power-to-weight ratio (W/kg) in endurance disciplines that are characterised by several climbs and descents. Taking the physiological variables that best correlated with the total race time, we performed a multiple regression analysis, aiming to predict the race performance in a UCI official competition. As a result, we found a significant regression equation that was able to explain 82% of the variance in performance from only three variables: PO_X4mmol/L_, PO_X2mmol/L_, and PO_VO2max_. These findings are not trivial since single regression models have shown that VO_2max_ can only explain 20–40% of the variance in cross-country cycling performance [7,8,15]. Thus, it seems that performance in mountain biking depends not on single but rather on multiple physiological features. Thus, the combination of maximal aerobic fitness variables as well as aerobic-anaerobic transition variables may represent the best ones to predict cross-country race performance, at least in young cyclists.

Finally, we compared the physiological profile of our cyclists with that of elite and young cross-country cyclists reported in previous studies (Table 3). According to our results, national-level youth cyclists have a suboptimal development of aerobic power and oxidative capacity compared to elite cross-country cyclists (>20 years old). Specifically, the PO_VO2max_ (in W/kg) in our young cyclists was up to 17% lower than in elite cross-country cyclists [4,5,6,7,8,10,11,31]. In addition, the PO_LT2_ (in W/kg) was equivalent to 66% of the PO_VO2max_ in our cyclists, while this threshold corresponded to 80–87% of the PO_VO2max_ in elite cross-country cyclists [5,8,10]. We also compared our results with those of the study by Fornasiero et al. [36] in top-level young cross-country cyclists of the same age as our cyclists. (~16 years old). We found that our young cyclists had a 21% lower PO_VO2max_ (in W/kg) than the Italian young cross-country cyclists. This might be explained by the fact that the athletes from the study by Fornasiero et al. were of a higher level than ours at that time. In fact, some of them were Youth Olympic and World Junior Champions, and their power profiles were even higher than that of elite cross-country cyclists.

### 4.3. Strength Profile

Most studies carried out on cross-country cyclists lack strength assessments, despite the fact that it is one of the most important variables in sports performance. In this respect, two recent studies performed multiple regression analyses and found that both handgrip strength and quadriceps femoris maximal torque were significant physiological determinants of cross-country cycling performance [1,13]. However, in the current study, none of the variables associated with the strength profile correlated with race time. In this study, we observed a significant correlation between the height obtained in a squat jump and P_sprint._ Thus, our results suggest that some lower-limb strength indices might act as independent predictors of short-duration maximal neuromuscular performance (i.e., P_sprint_) in youth cross-country cyclists, but it seemed that maximal jump height and maximal isometric strength in the lower limbs are not associated with mountain bike performance.

### 4.4. Limitations

Asides from its strengths, the current investigation presents some limitations that must be discussed so as not to affect the applicability of the results. First, the sample was composed of ten cyclists from the Argentinian National Junior Team of mountain biking. Although a higher sample size could have increased the statistical power of the study, the fact is that all the individuals of the National Team were recruited for the study. Further research with young cross-country cyclists from other National Teams may be required to confirm the importance of maximal and submaximal aerobic variables for overall mountain bike performance. Second, there were no measurements of ventilatory parameters such as VO_2max_ in the current investigation. This represents a limitation because this variable is a typical key indicator of mountain bike performance. However, we measured the POVO_2max_, which is also a reliable indicator of maximal aerobic capacity. Lastly, there are other variables that may be associated with mountain bike performance during real competitions, such as cycling economy, anaerobic power and capacity, technical ability as well as pre- and in-competition nutritional strategies [2]. Therefore, future investigations should be carried out to determine the contribution of each one of these variables to overall mountain bike performance.

## 5. Conclusions

This study provides coaches with valuable information about reference values of body composition, physiological markers of aerobic fitness, and neuromuscular performance in top-level youth cross-country cyclists. However, the most important contribution of this study was that the combination of the values of cycling power output at 2 mmol/L and 4 mmol/L of the blood lactate concentration and the cycling power at VO_2max_ was able to explain 82% of the variance in total race time in a group of top-level youth cross-country cyclists. In this regard, the results of this study support the use of maximal and submaximal indices of aerobic power and capacity as predictors of performance in youth populations who compete in mountain biking. These physiological parameters should be normalised according to body mass since this adjustment provides a better reflection of the athletes’ performance capacity. Finally, we also recommend the use of lower-limb strength assessments (e.g., squat jump height) as markers of neuromuscular performance in short-duration maximal exercises in young cyclist populations, although their predictive value on the overall mountain bike performance seems to be limited.

## Figures and Tables

**Table 1:** Anthropometric profile of elite youth cross-country cyclists.

Variable (Units)	Absolute Values	Relative Values (%)
Age (yr)	16.3 ± 0.95	-
Height (cm)	176.74 ± 4.23	-
Body Mass (kg)	67.22 ± 7.49	-
Muscle Mass (kg)	31.89 ± 5.56	47.25 ± 3.24
Adipose Mass (kg)	15.26 ± 1.43	22.91 ± 2.6
Bone Mass (kg)	9.11 ± 1.28	13.64 ± 1.95
Residual Mass (kg)	7.3 ± 1.17	10.84 ± 0.71
Skin Mass (kg)	3.75 ± 0.26	5.61 ± 0.35
Σ 6 Skinfolds (mm)	47.71 ± 8.24	-

Values are means ± SD.

**Table 2:** Physiological and strength profile of elite youth cross-country cyclists.

Variable (Units)	Absolute Values	Normalised Values (W/kg or N/kg)
PO_LT1_ (W)	187.0 ± 28.79	2.79 ± 0.41
PO_LT2_ (W)	232.75 ± 29.54	3.48 ± 0.41
PO_X2mmol/L_ (W)	217.24 ± 36.93	3.24 ± 0.49
PO_X4mmol/L_ (W)	258.34 ± 32.4	3.86 ± 0.43
PO_Dmax_ (W)	228.44 ± 21.96	3.41 ± 0.29
PO_VO2max_ (W)	354.6 ± 37.81	5.29 ± 0.38
CP (W)	262.9 ± 53.79	3.91 ± 0.65
P_10min_ (W)	286.9 ± 50.97	4.27 ± 0.58
P_5min_ (W)	319.2 ± 50.1	4.74 ± 0.51
P_1min_ (W)	499.3 ± 49.76	7.44 ± 0.26
P_30s_ (W)	704.1 ± 117.09	10.45 ± 0.94
P_sprint_ (W)	901.6 ± 178.18	13.52 ± 2.8
Squat Jump (cm)	26.6 ± 3.72	-
Right Knee Extension (N)	3827.1 ± 900.3	56.59 ± 8.39
Left Knee Extension (N)	3274.6 ± 1849.9	60.34 ± 7.74
Knee Extension Asymmetry (N)	493.8 ± 389.2	7.18 ± 4.96

Values are means ± SD. PO_LT1_ = power output at lactate threshold 1; PO_LT2_ = power output at the lactate threshold 2; PO_X2mmol/L_ = power output at a fixed blood lactate concentration of 2 mmol/L; PO_X4mmol/L_ = power output at a fixed blood lactate concentration of 4 mmol/L; PO_Dmax_ = power output at a lactate threshold calculated by the D_max_ method; PO_VO2max_ = power output at VO_2max_; CP = critical power; P_10min_ = mean power output during a 10 min exercise; P_5min_ = mean power output of a 5 min exercise; P_1min_ = mean power output during a 1 min exercise; P_30s_ = mean power output during a 30 s exercise; P_sprint_ = mean power output during a 6 s exercise.

**Table 3:** Anthropometric and power profiles of the national-level youth cross-country cyclists (white rows) and the data of elite cross-country cyclists reported in the literature (grey rows).

Study	Category	n	Age (Yr)	Height (cm)	Mass (kg)	PO_VO2max_ (W)	PO_VO2max_ (W/kg)	PO_LT2_ (W)	PO_LT2_ (W/kg)	PO_LT1_ (W)	PO_LT1_ (W/kg)	PO_Dmax_ (W)	PO_Dmax_ (W/kg)
Impellizzeri et al. [7]	Elite	12	25 ± 3	176 ± 7	66 ± 6	426 ± 40	6.4 ± 0.6	-	-	-	-	-	-
Lee et al. [6]	Elite	7	24 ± 3	178 ± 7	65 ± 7	413 ± 36	6.3 ± 0.5	-	-	-	-	339 ± 31 (82.1%)	5.2 ± 0.6 (82.5%)
Impellizzeri et al. [8]	Elite	13	20 ± 1	177 ± 8	65 ± 6	392 ± 35	-	340 ± 38 (86.7%)	-	286 ± 32 (72.9%)	-	-	-
Stapelfeldt et al. [10]	Elite	9	21 ± 2	180 ± 6	69 ± 5	368 ± 25	5.3 ± 0.3	295 ± 25 (80.1%)	-	215 ± 24 (58.4%)	-	-	-
Warner et al. [31]	Elite	16	26 ± 5	178 ± 5	71 ± 5	-	-	-	-	-	-	-	-
Impellizzeri et al. [5]	Elite	5	21 ± 4	175 ± 3	65 ± 5	367 ± 36	5.7 ± 0.6	318 ± 14 (86.6%)	4.9 ± 0.4 (86.0%)	276 ± 17 (75.2%)	4.3 ± 0.2 (75.4%)	-	-
Baron [4]	Elite	25	23 ± 4	179 ± 5	69 ± 7	-	5.5 ± 0.4	-	-	-	-	-	-
Wilber et al. [11]	Elite	10	29 ± 4	176 ± 7	72 ± 8	420 ± 42	5.9 ± 0.3	-	-	-	-	-	-
Fornasiero et al. [36]	Junior	12	16 ± 0	173 ± 7	60 ± 5	395 ± 41	6.7 ± 0.6	-	-	-	-	-	-
Present study	Junior	10	16 ± 1	177 ± 4	67 ± 7	355 ± 38	5.3 ± 0.4	233 ± 30 (65.6%)	3.5 ± 0.4 (66%)	187 ± 29 (52.7%)	2.8 ± 0.4 (52.8%)	228 ± 22 (64.2%)	3.4 ± 0.3 (64.2%)

## Data Availability

The data presented in this study are available on request from first author of this study.
